# Ectopic Intratracheal Thyroid: A Rare Cause of Airway Obstruction

**DOI:** 10.1155/2018/2897943

**Published:** 2018-01-27

**Authors:** Waheed Rahman, Nafil Ishaq Arimbrathodi, Faisal Abdulkader, Hussain Al-Enazi, Zeynel A. Dogan

**Affiliations:** Department of Otolaryngology, Head and Neck Surgery, Hamad Medical Corporation, Doha, Qatar

## Abstract

Ectopic intratracheal thyroid tissue (EITT) is a rare abnormality with only limited cases reported so far. The presenting symptoms can be very similar to those of bronchial asthma. We discuss the case of a 29-year-old man with subglottic ectopic thyroid, with a history of thyroid surgery for goiter, which has been managed with laser-assisted endoscopic approach. We have also included presenting symptoms, pathophysiology, diagnosis, and management of EITT. We aim to include EITT in the differentials of airway obstruction, particularly in those patients who have goiter or previous thyroid surgeries.

## 1. Introduction

Ectopic thyroid tissue (ETT) is a rare entity, which can be seen anywhere in the midline from the base of the tongue to mediastinum. Among them, the laryngotracheal region has the rarest occurrence that can cause upper airway obstruction. Since ectopic intratracheal thyroid tissue (EITT) is a rare condition, its symptoms can easily be mistaken for those of asthma. An associated hypothyroid level can lead to enlargement of EITT and worsening of symptoms. We are discussing EITT-causing stridor in a young man with a history of thyroid surgery who missed thyroxine therapy.

## 2. Case Report

A 29-year-old male, nonsmoker, presented to the emergency department with breathing difficulty. He had increasing dyspnea since few months, for which his general practitioner had treated him for asthma. Five years earlier, the patient had undergone thyroid surgery for goiter with subsequent thyroxine replacement therapy. But the patient missed the course and follow-up.

During the current presentation, fiber optic endoscopy showed bilateral mobile vocal cords and narrow infraglottic trachea with a right-sided subglottic mass.

**Figure 1:**
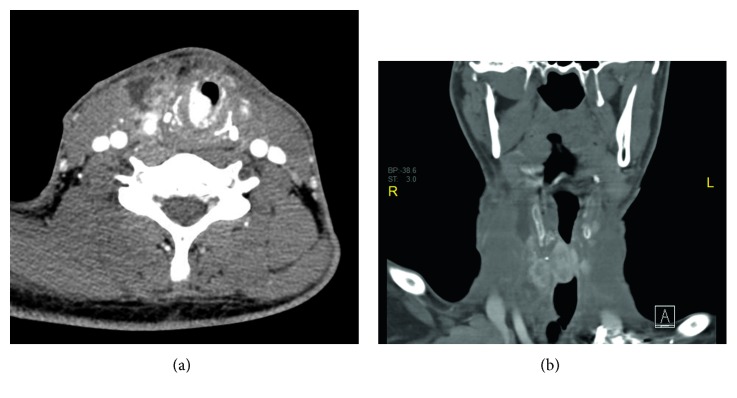
Contrast-enhanced CT showing subglottic soft tissue mass extending at the level of C4, C5, C6, C7 vertebrae, causing marked narrowing of the airways. (a) is axial view and (b) is coronal view.

CT showed subglottic soft tissue mass of about 4 × 4.7 × 2.4 cm in the craniocaudal, anteroposterior, and transverse dimensions, respectively, extending at the level of C4, C5, C6, and C7 vertebrae, and it causes marked narrowing of the airways (Figures 1(a) and 1(b)).

On admission, TSH was 7.82 mIU/L (normal range 0.5–5.0 mIU/L) and free T4 was 13.31 mcg/dl (normal range 7.0–14.0 mcg/dl).

**Figure 2:**
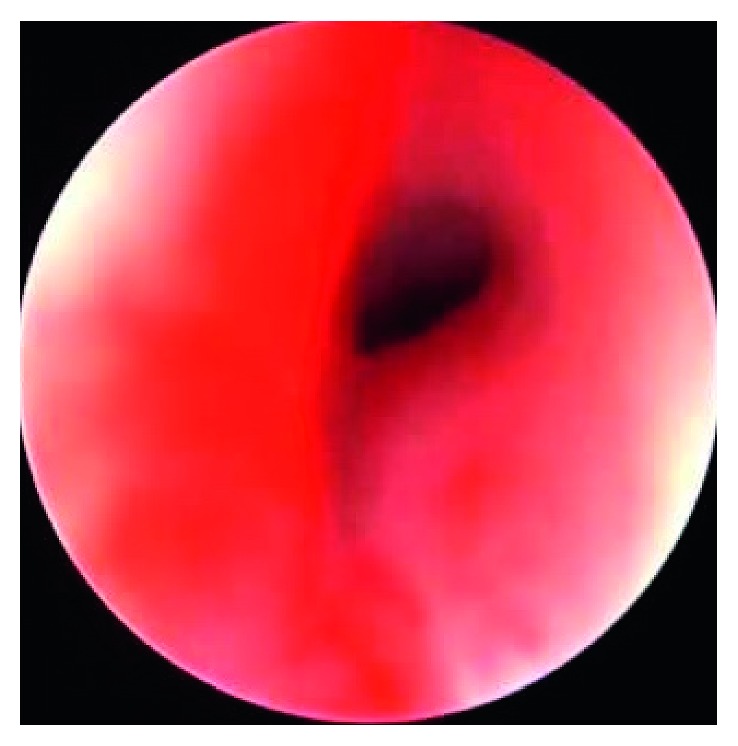
Tracheo-laryngoscopic examination showing subglottic mass obstructing the airway.

The patient underwent emergency tracheobronchoscopy which showed subglottic mass (Figure 2). Biopsy of mass done and the patient was tracheostomised. The histopathology and immunostain study result confirmed multinodular goiter associated with hyperplastic nodules with no evidence of malignancy.

**Figure 3:**
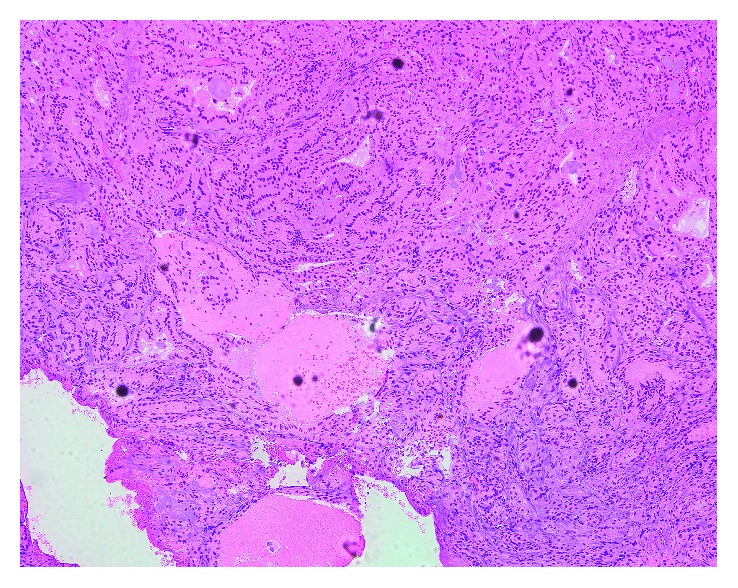
Histopathology showing thyroid follicles filled with colloid.

**Figure 4:**
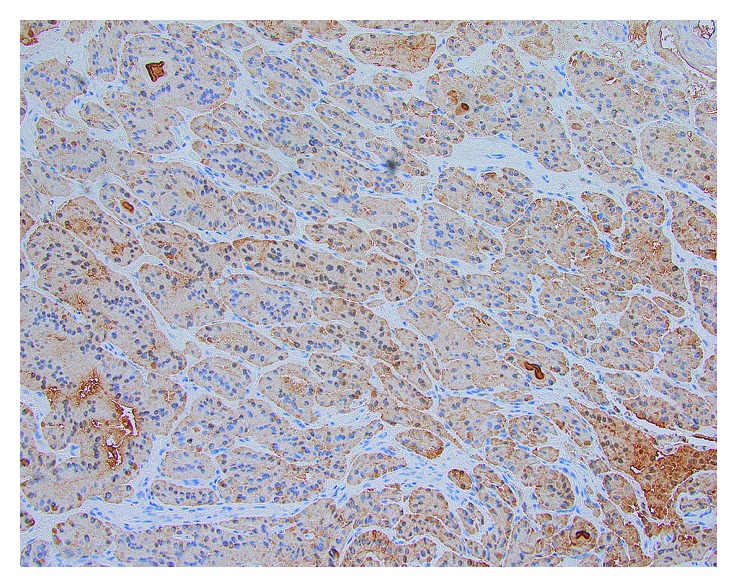
Positive cytoplasmic staining with thyroglobulin immunostain confirming the thyroid origin.

**Figure 5:**
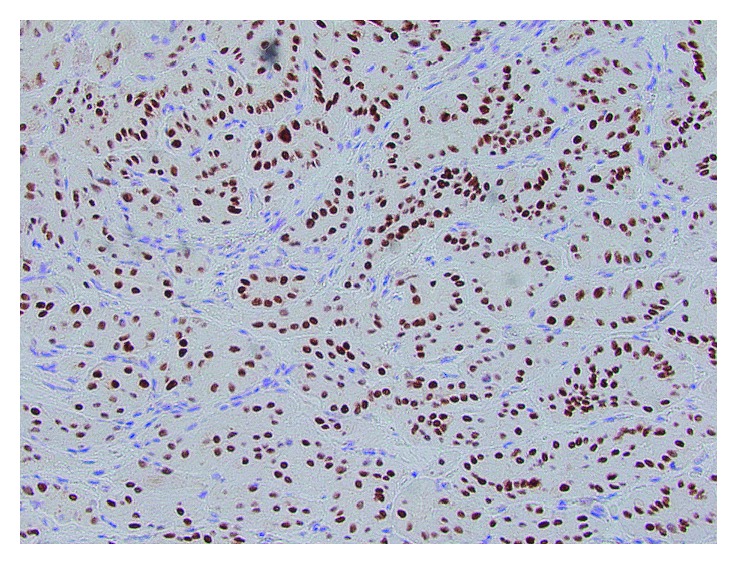
Positive nuclear staining with TTF-1 immunostain confirming the thyroid origin.

We have done microlaryngoscopic excision of the subglottic mass utilizing CO_2_ laser. The histopathology confirmed ectopic thyroid tissue (Figures 3–5). The patient weaned from tracheostomy tube and discharged on thyroxin supplement, and regular follow-up with ENT and endocrinology was given.

## 3. Discussion

Being a rare phenomenon, the number of cases reported with ETT so far is less than 500 [1, 2]. It is most commonly found in the lingual, sublingual, thyroglossal, laryngotracheal, and lateral areas. Very rarely, it has been described at the esophagus, mediastinum, heart, pancreas, adrenals, small intestine, and cutaneous area [3–12].

The percentage of ETT in trachea contributes 6–7% of all primary endotracheal tumors [13]. The first case was described in 1875 by Ziemssen [14–16]. The majority of the cases were reported from endemic goiter regions of the world [17]. Though EITT has been found anywhere from glottis to tracheal bifurcation, its most common appearance is as a submucosal mass on the lateral subglottic and upper tracheal wall [17–19].

The origin of EITT is explained by two theories. “The malformation theory” by Ziemssen in 1875 postulates that tracheal cartilage which develops later splits the thyroid gland, creating a small ectopic nest in the tracheal cavity [15, 16]. “The ingrowth theory” by Paltauf in 1892 states that the improper development of the mesenchymal tissue between the thyroid and trachea results in adherence of the former to the latter thus permitting the ingrowth [20].

Usually, patients are symptomless until a change in their hormonal status happen. Stimulatory factors including TSH, epidermal growth factor, and human chorionic gonadotropin could stimulate thyroid growth [21]. Symptomatic EITT causes difficulty in breathing, cough, stridor, and dysphagia. On physical examination, differentiating stridor of EITT from wheezing of asthma is not easy.

The findings suggestive of malignancy of EITT include multiple nodules, ulceration, and bleeding. 11% of EITT has been reported to undergo malignant transformation [16]. The most common malignancy found is papillary thyroid carcinoma [22, 23].

In a symptomatic patient, complete ENT examination should be carried out. Laboratory studies including thyroid assessment should also be conducted. Indirect laryngoscopy, flexible laryngoscopy, and CT and MR studies must be included. Radionuclide studies are highly sensitive and specific in detecting functional EITT [24]. Tc-99m pertechnetate scans high value in assessing the size, distribution, and activity of EITT. Fusion imaging techniques like single photon emission tomography-computed tomography (SPECT-CT) is valuable in diagnosis of EITT.

If associated with multinodular goiter, FNAC can be done. Direct laryngobronchoscopy will help in proper visualization of mass and performing biopsy. Since there is a chance of severe hemorrhage, a biopsy should be carried carefully [16].

The factors determining management of EITT include the size of lesion, the patient's age, the presence of local symptoms, the status of thyroid function, and histological findings. Surgical excision, radioiodine ablation, and thyroid suppression therapy are the main treatment options adapted. The complete surgical excision is indicated in symptomatic cases and in cases of histological malignancy. The two common surgical methods are removal via an endoscopic laser-assisted approach as in the present case and removal of the tumor via an open cricoid procedure [25]. Postoperative thyroid suppression therapy is performed to prevent hypertrophy of residual tissue. Radioiodine ablation with hormonal suppression has only limited success [25]. The risks associated with ablation therapy include radiation thyroiditis and tracheitis. It can be useful in patients who refuse or are unfit for surgery. While considering all the management options, surgical removal should be counted as the best option for the long-term outcomes [26–28].

## 4. Conclusion

Even though EITT is a rare abnormality with few case reported in the literature, it must be included in the differentials in patients presenting with airway obstruction, particularly in those who have goiters or previous thyroid surgeries.
